# Editorial: Digital health and virtual health care for adults and older adults: innovative technological solutions for diagnosis, management, and rehabilitation

**DOI:** 10.3389/fmed.2024.1417069

**Published:** 2024-05-21

**Authors:** Davide Maria Cammisuli, Paolo Taurisano, Björn Wolfgang Schuller, Kun Qian

**Affiliations:** ^1^Department of Psychology, Catholic University of the Sacred Heart, Milan, Italy; ^2^Department of Translational Biomedicine and Neuroscience, Aldo Moro University of Bari, Bari, Italy; ^3^Imperial College London, London, United Kingdom; ^4^University of Augsburg, Augsburg, Germany; ^5^Beijing Institute of Technology, Beijing, China

**Keywords:** digital health, technological devices, aging, adults, older adults

The social and health costs of caring for the elderly are high due to frailty, polypathology, and disability derived from cardiovascular, neurological/psychiatric diseases, with a significant improvement after the COVID-19 pandemic (1), so the new watchword is “aging in place.” Technological solutions to reduce dependency on caregivers are now commonplace (2). Digital technology is rapidly transforming healthcare for adults and older adults, through devices, sensors, IoT solutions, services, and programs that can lead to improved clinical awareness and therapeutic advances. The development of safe, reliable, and intelligent medical devices or wearable devices at sustainable costs makes healthcare accessible to people and improves around the world (3).

In this Research Topic, we have focused on manuscripts that present novel insights into innovative technological solutions for the diagnosis, management, and rehabilitation of adults and older adults, in order to provide this Journal's readers with an updated and comprehensive scenario of the latest advances in scientific research and to inform health care systems.

An online study by Wrede et al. on contactless monitoring technology reports a reliable reference framework for clinicians and researchers aiming to investigate the views of caregivers about cognitively impaired people living alone. The main strength of this article was the fact that it was conceived in two different European countries (i.e., the Netherlands and Germany), which increased the chance of achieving a fit between technology, users, and use contexts.

In a scoping review that included results from studies conducted in different countries (i.e., the United States, China, Italy, South Korea, Greece, the United Kingdom, Japan, Thailand, Sweden, and Australia), a similar conclusion was that it highlighted the usability of sensor-based technology to identify cognitive changes over time or to detect behavioral changes in the elderly in assisted living solutions. With the aim of expanding. scientific knowledge about low-income countries, the institutional-based cross-sectional study by Walle et al. had the merit of shedding light on Ethiopia's national health information system, which incorporates clinical data at different levels to improve the delivery of healthcare services and decision-making in health care policy.

With regard to the most advanced biomedical technologies, Zhong et al. claimed that oblique lumbar interbody fusion (OLIF) is becoming increasingly widespread in the treatment of degenerative diseases of the lumbar spine due to its marginal invasiveness, indirect decompression, high fusion speed and fast rehabilitation. In their scientific work, the authors carefully described the construction of the full L1–L5 lumbar model, the development of an OLIF surgical model, and compressive pressure analysis to understand the crucial role in strengthening the lumbar spine's load-bearing capacity.

The clinical trial by Siggaard et al. reported the feasibility of a new method of digital screening for ear-nose-and-throat (ENT) specialist evaluation in a sample of hearing-impaired adults without previous hearing aid usage or experience, limiting the risk of misdiagnosis. This method is particularly suitable for large-scale evaluation of such clinical populations. Chapman-Goetz et al. (2023) conducted a prospective single-blind randomized controlled trial demonstrating that a health management app for tracking medications and other important health features is an achievable tool for providing treatment adherence in patients with cronic heart failure. Moreover, D'Ambrosio et al. implemented a research protocol on the risks of fall in the elderly through the use of the DigiRehab platform for physical rehabilitation at home, whose main focus consists of providing preventive screening, assignment of tailored exercises, and training for caregivers. Through a single-arm prospective study, another research group coordinated by D'Silva et al. documented that a gaming app developed for vestibular rehabilitation (i.e., the VestRx) improves performance accuracy, involvement in exercises, and error correction in patients with unilateral or bilateral vestibular hypofunction, due to vestibular neuritis, labyrinthitis or Meniere's disease.

A very recent application of technological solutions for the digital care of older adults, such as smart sensory garments, can be used to monitor neurophysiological activation related to spatial cognition (SC) in patients suffering from Mild Cognitive Impairment due to Alzheimer's disease (MCI due to AD) (4). Through an ecological paradigm capable of evaluating SC behavioral disorders in the elderly along the continuum of normal aging to Alzheimer's type dementia, we have shed light on the activation of the autonomic nervous system related to the topographical disorientation that characterized patients with prodromal AD while walking unfamiliar routes (5). Specifically, participants wore a smart sensory garment (i.e., the Comftech Howdy Senior^®^, Monza, Italia) able to provide an instant report, displayed on the connected smartphone App, on cardiac parameters (among which the RMSSD, as the root mean square of successive differences between normal heartbeats), respiratory parameters (among which the SDBR, as the standard deviation of cumulative breathing rate), and accelerometer parameters during a naturalistic walking task of spatial navigation (i.e., the Detour Navigation Test-modified version) performed in an urban public garden (5). We tested 64 participants (*mean age* = 72.23 ± 6.42; education = 11.84 ± 3.89; M = 34; F = 30), among them 22 patients with MCI due to AD, 22 individuals with subjective cognitive decline (SCD), and 20 healthy controls (HCs). In the subgroup of patients with MCI due to AD, we observed a failure of the egocentric sequential learning as evidenced by more wrong turns (WTs) in the route retracing task (in numbers) than in individuals with SCD (Mann–Whitney *U*-test = 72.500, *p* = 0.001, η^2^ = 0.39) and healthy controls (Mann–Whitney *U*-test = 57.000, *p* = 0.001, η^2^ = 0.42; WTs in number, MCI due to AD = 94; SCD = 32; HCs = n. 22). The comparison between the SCD and HCs subgroups on this variable was not significant (*p* = n.s.). In turn, higher WTs were negatively associated with cardiac parameters (RMSSD, rho = −0,481, *p* > 0.05), and positively associated with respiratory parameters (SDBR, rho = 0.460; *p* < 0.05) only in the MCI due to the AD subgroup.

Such a finding revealed neurophysiological activation in patients with prodromal AD experiencing topographical disorientation, which can be specifically captured thanks to wearable textile instruments capable of continuously recording the patient's physiological data linked to cognitive impairment, without interfering with their naturalistic walking. Remarkably, a lower parasympathetic system self-regulation of an increasing number of breaths correlated with higher topographic disorientation, respectively.

These results could be used to establish increasing physiological thresholds (e.g., low/medium/-high) indicating variations in tonic alertness, in order to prevent spatial disorientation when cognitively impaired people living in the community are requested to leave their homes. A smart technological apparatus, like a sensory garment and a smartphone with a dedicated App, may be able to alert clinicians/caregivers to the presence of dangerous physiological patterns in adults and older adults with potential risk factors thanks to remote backend servers with a control dashboard, and to provide them with immediate digital feedback.

## Author contributions

DMC: Writing – original draft. PT: Writing – review & editing. BS: Writing – review & editing. KQ: Writing – review & editing.

## References

[1] Schuller BW Pham PN Do S Pattichis CS Nair P editors. Health Technologies and Innovations to Effectively Respond to the COVID-19 Pandemic. Lausanne: Frontiers Media SA (2022). 10.3389/fdgth.2022.849652 PMC8884238 35237764

[2] D'Amico F Lancioni GE De Marinis F Abbinante F Taurisano P Abbatantuono C. A technology-aided program to support positive occupation in people with advanced Alzheimer's disease: a pilot study. Technol Disabil. (2023) 4:1–8. 10.3233/TAD-170182

[3] Qian K Schuller BW Guan X Hu B. Intelligent music intervention for mental disorders: insights and perspectives. IEEE Trans Comput Soc Syst. (2023) 10:2–9.20492059

[4] Cammisuli DM Isella V Verde F Silani V Ticozzi N Pomati S. Behavioral disorders of spatial cognition in patients with mild cognitive impairment due to Alzheimer's disease: preliminary findings from the BDSC-MCI project. J Clin Med. (2024) 13:1178. 10.3390/jcm13041178 38398490 PMC10889220

[5] Cammisuli DM Tuena C Riva G Repetto C Axmacher N Chandreswaran V. Exploring the remediation of behavioral disturbances of spatial cognition in community-dwelling senior citizens with mild cognitive impairment via innovative technological apparatus (BDSC-MCI Project): protocol for a prospective, multi-center observational study. J Pers Med. (2024) 14:192. 10.3390/jpm14020192 38392625 PMC10890288

